# The Authors’ Reply

**Published:** 2014-05

**Authors:** Sayed Mohammad Reza Hadavi, Elaheh Allahyary, Saman Asadi

**Affiliations:** Anesthesiology and Critical Care Research Center, Department of Anesthesiology, Faghihi Hospital, Shiraz University of Medical Sciences, Shiraz, Iran

## Dear Editor,

We thank Dr Cascella for his insightful comments and the opportunity to clarify a number of points from our work.

Anesthesia is not a single pharmacologic process. Rather, it is a complex interaction of multiple stimuli, diverse responses and drug-induced probability of non-responsiveness to stimuli. Anesthesia is defined by its hypnotic (unconsciousness) and analgesic (pain relief) components. The hypnotic effects of the intravenous and inhaled anesthetics can be measured with empirically derived indices calculated from an EEG, such as the Bispectral Index (BIS).^1^ However BIS may not be a gold standard monitor for the evaluation of all components of anesthesia depth. There is emerging evidence that intra-operative monitoring of the hypnotic component of an anesthetic regimen may decrease the risk of awareness associated with anesthesia. Perhaps it is better to state that we have used BIS to evaluate the adequacy of the hypnotic component of our general anesthesia regimen in C/S patients.

In response to your opinion about our small sample size for evaluation of awareness, it is exactly acceptable. However, it was not our intent to study the incidence of awareness in these cases. We have only collected and reported awareness as non-conclusive data. Additionally, we asked our patients to inform us about any probable recollections over the days following discharge from the hospital. In this regard, we did not receive any ongoing data.

We are in complete agreement with your explanations regarding explicit memory, consciousness, recall, awareness and post-traumatic stress syndrome.

In the near future, we hope to attain new technologies and monitoring systems for complete, accurate evaluation of anesthesia depth.
